# Successful treatment of a full-thickness mucosal injury with a fully covered metal stent during peroral endoscopic myotomy

**DOI:** 10.1055/a-2615-6134

**Published:** 2025-06-26

**Authors:** Anna Pèlach, Luis Wong, Raquel Muñoz-González, Elisenda Garsot, Harold Benites-Goñi, Vicente Moreno de Vega, Hugo Uchima

**Affiliations:** 1 16514 Endoscopy Unit, Gastroenterology Department, Hospital Universitari Germans Trias i Pujol, Badalona, Barcelona, Spain; 2 16711 Endoscopy Unit, Centro Médico Teknon, Barcelona, Spain; 3 Research Staff, Fundació Institut d’Investigació en Ciències de la Salut Germans Trias i Pujol, Badalona, Spain; 4 16514 Esophagogastric Surgery Unit, Hospital Universitari Germans Trias i Pujol, Badalona, Spain; 5 33225 Vicerrectorado de Investigación, San Ignacio de Loyola University, Lima, Peru

Peroral endoscopic myotomy (POEM) is a highly effective and safe first-line treatment for achalasia 1 . The rate of full-thickness mucosal injuries during POEM has been reported at 1.7%, occurring most commonly in the cardia 2 . Significant risk factors associated with full-thickness mucosal injuries include previous POEM (OR, 5.005) and submucosal fibrosis (OR, 12.074) 2 . Small mucosal injuries are usually easily treated using endoscopic clips, whereas larger injuries may require additional techniques such as suturing or stents 2 . Since achalasia lacks peristalsis, stent migration risk may be lower.

We present the case of a 23-year-old man who underwent POEM for type II achalasia. Although previous biopsies showed four eosinophils per high-power field, this finding was not conclusive for eosinophilic esophagitis.

During POEM, inadvertent intramuscular tunneling left muscle fibers attached to the mucosal plane, requiring further dissection and potentially compromising the muscularis mucosae ( Video 1 ). A spastic area was identified, and a thin pediatric gastroscope was used for double-scope transillumination 3 , requiring a forced push to advance it, and an unusual translucency suggestive of mucosal injury was seen ( Fig. 1 ). Intraluminal evaluation confirmed mild mucosal changes, prompting initiation of myotomy to prevent further damage.

Successful treatment of a full-thickness mucosal injury during peroral endoscopic myotomy with a fully covered metal stent.Video 1

**Fig. 1:**
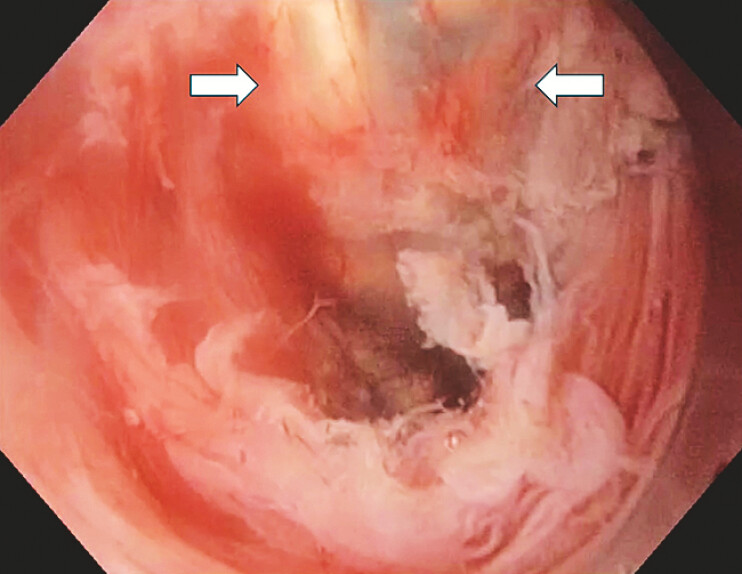
Unusual translucency (arrows) during double-scope transillumination.

After the myotomy was initated, a second intraluminal inspection revealed a full-thickness mucosal injury ( Fig. 2 ). To complete the procedure safely, underwater myotomy was performed. Upon completion of the myotomy, attempts to close the defect with clips were unsuccessful due to mucosal denudation and direct communication with the mediastinum. Finally, after closure of the mucosal incision with clips, a fully covered metal stent was placed to seal the defect ( Fig. 3 ). A nasogastric tube was subsequently inserted under endoscopic guidance, and prophylactic antibiotics were administered. A computed tomography scan ruled out any leaks, allowing the patient to begin oral intake and be discharged after one week without complications. The stent was removed three weeks post-procedure, and complete re-epithelialization was confirmed.

**Fig. 2:**
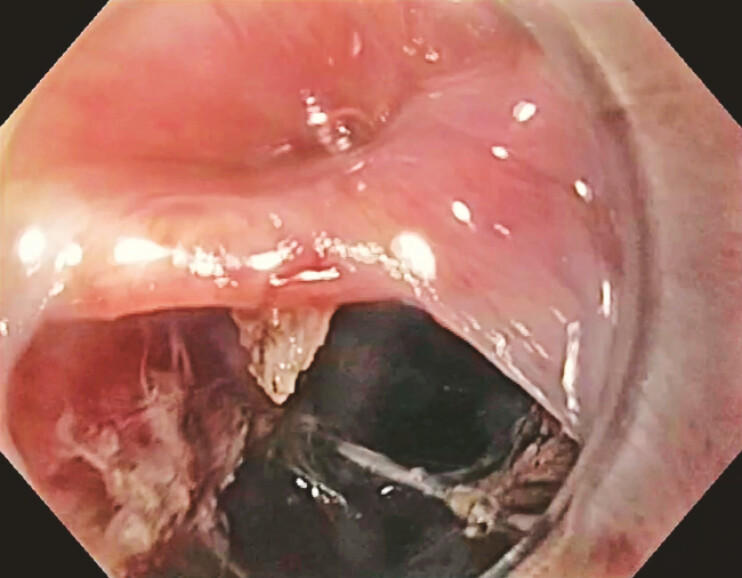
Full-thickness mucosal defect.

**Fig. 3:**
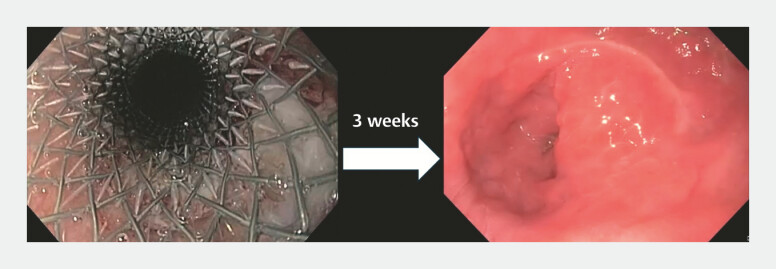
Fully covered metal stent placed for the full-thickness defect (left). Complete re-epithelization after removal of the stent three weeks post-procedure (right).

Endoscopy_UCTN_Code_CPL_1AH_2AL

## Notes

Endoscopy E-Videos https://eref.thieme.de/e-videos
*E-Videos* is an open access online section of the journal *Endoscopy* , reporting on interesting cases and new techniques in gastroenterological endoscopy. All papers include a high-quality video and are published with a Creative Commons CC-BY license. Endoscopy E-Videos qualify for HINARI discounts and waivers and eligibility is automatically checked during the submission process. We grant 100% waivers to articles whose corresponding authors are based in Group A countries and 50% waivers to those who are based in Group B countries as classified by Research4Life (see: https://www.research4life.org/access/eligibility/ ). This section has its own submission website at https://mc.manuscriptcentral.com/e-videos .
